# Profiling Genetic Variation: Divergence Patterns and Population Structure of Thailand’s Endangered *Celastrus paniculatus* Willd

**DOI:** 10.3390/biology14060725

**Published:** 2025-06-19

**Authors:** Kornchanok Kaenkham, Warayutt Pilap, Weerachai Saijuntha, Sudarat Thanonkeo

**Affiliations:** 1 Graduate School, Mahasarakham University, Maha Sarakham 44150, Thailand; kornchanok.b@ubu.ac.th; 2 Walai Rukhavej Botanical Research Institute, Mahasarakham University, Maha Sarakham 44150, Thailand; warayutt@msu.ac.th; 3 Center of Excellence in Biodiversity Research, Mahasarakham University, Maha Sarakham 44150, Thailand; weerachai.s@msu.ac.th; 4 Faculty of Medicine, Mahasarakham University, Maha Sarakham 44000, Thailand

**Keywords:** genetic divergence, population structure, plant conservation, molecular phylogenetic, nuclear and plastid markers, ITS and *rbcL* sequence analyses

## Abstract

This study examined genetic diversity in the endangered medicinal plant *Celastrus paniculatus* using 62 individual samples from seven natural populations in northern and northeastern Thailand to inform conservation strategies. The analysis of the nuclear internal transcribed spacer (ITS) and ribulose-1,5-bisphosphate carboxylase/oxygenase large subunit (*rbcL*) markers revealed 17 haplotypes (CpI1–CpI17) across these populations, with 15 being population-specific. The genetic diversity varied significantly among populations: CMI showed the highest diversity (Hd = 0.944 ± 0.070), while LEI and LPN displayed complete homogeneity. The haplotype network identified a central shared haplotype (CpI4), suggesting a common ancestry, with the PLK population showing a distinct genetic divergence through unique haplotypes separated by multiple mutation steps. Genetic distance calculations revealed close relationships between LEI and NPM populations (distance = 0.0004), with greater differentiation between PLK and other populations (distances > 0.005). Phylogenetic analyses confirmed the species integrity while highlighting population clusters, especially PLK in ITS analyses and LPN in *rbcL* analyses. This genetic structure information provides a foundation for targeted conservation planning. Results suggest that conservation efforts should prioritize both genetically diverse populations (like CMI and MKM) and genetically distinct ones (like PLK) to preserve the maximum evolutionary potential. This study delivers crucial molecular data for developing evidence-based conservation strategies to protect this valuable medicinal species from further decline.

## Simple Summary

*Celastrus paniculatus*, also known as the “intellect tree,” is an important medicinal plant in Thailand valued for its neuroprotective and anti-inflammatory properties. Because this species is endangered, a study was conducted to analyze its genetic diversity to create a conservation plan. The research revealed significant genetic differences among plant populations in northern and northeastern Thailand; some had high diversity, while others were genetically very unique. The study concludes that conservation efforts must focus on protecting both the genetically diverse and the genetically distinct populations to ensure the long-term survival of this valuable plant.

## 1. Introduction

*Celastrus paniculatus* Willd. (Celastraceae), commonly known as Jyotishmati, Malkangani, or the “intellect tree”, is an evergreen woody climber with significant ethnomedicinal importance throughout its native range in Asia and Southeast Asia [1,2]. In Thailand, particularly in the northern and northeastern regions, this species has been traditionally utilized for its cognitive-enhancing properties, anti-inflammatory effects, and treatment of various ailments, including neurological disorders, rheumatism, and digestive complaints. The seeds contain numerous valuable bioactive compounds, such as alkaloids, flavonoids, and specific sesquiterpenes like celastrine, responsible for its demonstrated neuroprotective, antioxidant, and anti-inflammatory properties in pharmacological studies [2,3,4,5,6].

Despite its considerable medicinal value, *C. paniculatus* faces significant threats to its survival in Thailand. Habitat fragmentation due to agricultural expansion, over-harvesting for traditional medicine, and climate change have collectively contributed to its precarious situation. While a formal conservation assessment for *C. paniculatus* using IUCN Red List or Thailand Red Data List criteria has not yet been conducted for populations within Thailand, reports from Sri Lanka and India emphasize similar vulnerabilities, where the species is also considered threatened [7,8]. Given the increasing demand and unsustainable harvesting practices, *C. paniculatus* is at risk of becoming regionally endangered if conservation actions are not implemented promptly. The species now exists primarily in fragmented populations across northern and northeastern Thailand, raising concerns about genetic erosion and reduced adaptive potential. This situation necessitates the development of effective conservation strategies based on comprehensive genetic information to ensure the long-term survival of this valuable medicinal resource. Understanding the genetic diversity and population structure of endangered plant species is fundamental to designing appropriate conservation strategies. Genetic diversity represents the evolutionary potential of a species, enabling the adaptation to environmental changes and resilience against diseases and pests. The loss of genetic variation can lead to inbreeding depression, reduced fitness, and an increased extinction risk. For medicinal plants like *C. paniculatus*, genetic diversity may also correlate with variations in phytochemical profiles and therapeutic properties, making its conservation particularly important from both ecological and pharmacological perspectives.

Molecular markers have become invaluable tools for assessing genetic diversity and elucidating population structures in plant conservation genetics. Over the past decades, numerous molecular marker systems have been developed that offer advantages over traditional morphological and biochemical approaches [9,10]. These include the restriction fragment length polymorphism (RFLP), amplified fragment length polymorphism (AFLP), random amplified polymorphic DNA (RAPD), simple sequence repeat (SSR), sequence characterized amplified region (SCAR), inter-simple sequence repeat (ISSR), and single nucleotide polymorphism (SNPs), among others. For phylogenetic studies and population genetics of medicinal plants, DNA sequence-based markers have proven to be particularly informative. Among these, the nuclear internal transcribed spacer (ITS) region and the chloroplast ribulose-1,5-bisphosphate carboxylase/oxygenase large subunit (*rbcL*) gene complement each other effectively in revealing evolutionary patterns. The ITS region evolves relatively rapidly, providing insights into recent evolutionary events and fine-scale population structures, while the more conserved *rbcL* marker illuminates deeper evolutionary relationships and maternal lineage patterns in plants [10,11,12,13].

To date, comprehensive genetic studies of *C. paniculatus* in Thailand have been limited, creating a significant knowledge gap for conservation planning. Previous studies have primarily focused on the species’ phytochemical properties or have examined genetic diversity in other parts of its range, such as India. The lack of detailed genetic information specific to Thai populations hampers effective conservation efforts and sustainable utilization strategies for this valuable medicinal resource. This study aims to address this knowledge gap by investigating the genetic diversity and population structure of *C. paniculatus* across its distribution in northern and northeastern Thailand using both nuclear (ITS) and chloroplast (*rbcL*) DNA markers. The ITS region, known for its relatively high variability, is suitable for detecting intraspecific variations and population structures. In contrast, the more conserved *rbcL* gene provides complementary information from the chloroplast genome and is useful for broader taxonomic comparisons due to its universality and the availability of reference data [14]. By identifying patterns of genetic variation, unique haplotypes, and evolutionary relationships among populations, this research provides crucial information for developing targeted conservation strategies. The findings will contribute to preserving both the overall genetic diversity and evolutionarily distinct populations, thereby maintaining the species’ adaptive potential and ensuring the continued availability of this important medicinal plant for future generations.

## 2. Materials and Methods

### 2.1. Chemicals

The experimental materials were sourced from reputable suppliers to ensure reliability. The RBC genomic DNA kit was acquired from Life Biomedical Limited (Cambridge, UK), while Takara Ex *Taq* polymerase was sourced from Takara Bio Inc. (Shiga, Japan). For nucleic acid visualization, GelRed™ Nucleic acid gel stain from Biotium Inc. (Hayward, CA, USA) was utilized. The DNA purification kit (E.Z.N.A.^®^ gel extraction kit) was purchased from Omega Bio-Tek, Inc., Norcross, GA, USA. All solutions were prepared with nuclease-free water obtained from ITS (Thailand) Co., Ltd. (Bangkok, Thailand) to prevent contamination and maintain experimental integrity.

### 2.2. Sample Collection

**Figure 1:**
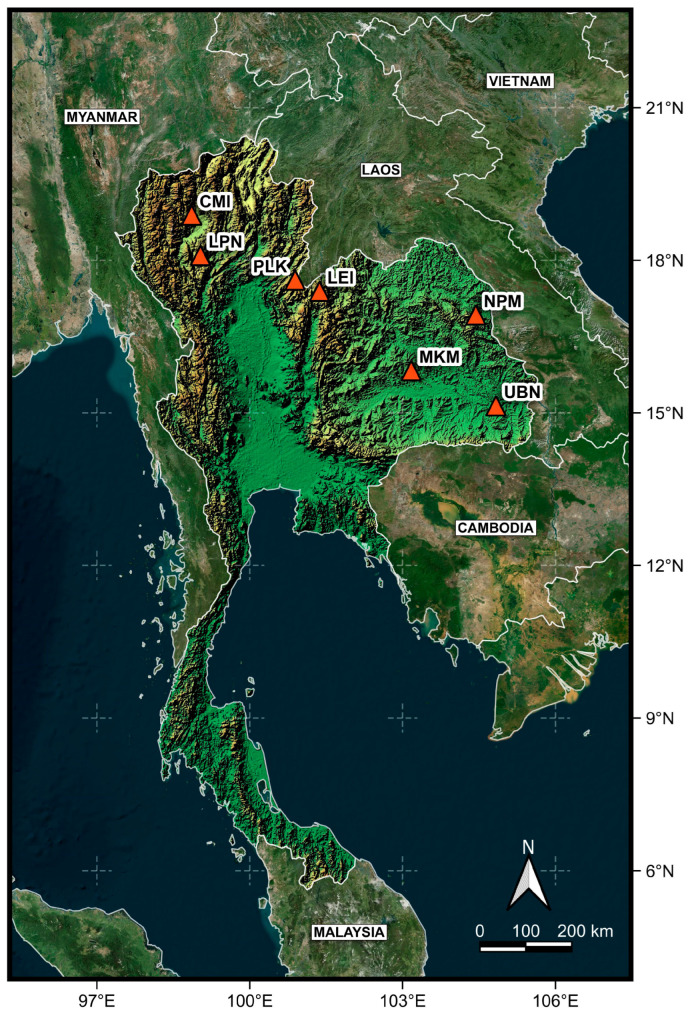
A map showing the geographic distribution of *Celastrus paniculatus* sampling locations in this study. Locality codes are provided in Table 1.

Young leaves and fruit capsules of *C. paniculatus* were collected from 62 mother trees across seven natural habitat locations in northern and northeastern Thailand, with the altitude, latitude, and longitude of each collection site carefully documented (Table 1 and Figure 1). After collection, leaves were preserved in silica gel before being transported to the laboratory at Walai Rukhavej Botanical Research Institute (WRBRI), Mahasarakham University, where voucher specimens were subsequently deposited. All collected capsules underwent formal identification and authentication by taxonomists at both the Queen Sirikit Botanic Garden in Chiang Mai and at WRBRI, following the established species identification key for the Celastraceae family [15].

### 2.3. Molecular Analysis

Genomic DNA was extracted from collected leaf samples using the RBC genomic DNA kit in accordance with the manufacturer’s protocol. All extracted DNA samples were maintained at −20 °C for preservation until subsequent analysis could be performed. For molecular characterization, both nuclear and plastid DNA regions were targeted. The nuclear internal transcribed spacer (ITS) region was amplified using the primer pair ITS-F (5′-AGA AGT CGT AAC AAG GTT TCC G-3′) and ITS-R (5′-TCC GCT TAT TGA TAT GCT TAA A-3′) as previously described by Wonnapinij and Sriboonlert [16]. For the plastid DNA, the ribulose-1,5-bisphosphate carboxylase/oxygenase large subunit (*rbcL*) gene was amplified using primers RbcL-F (5′-ATG TCAC CAC AAA CAG AGA CTA AAG C-3′) and RbcL-R (5′-GCA GCA GCTA GTT CCG GGC TCC A-3′) following protocols established by Hasebe et al. [17] and Asahina et al. [18].

Polymerase chain reaction (PCR) amplifications were conducted in 25 μL reaction volumes containing 16.375 μL of deionized H_2_O, 0.125 μL of Takara Ex *Taq* polymerase (5 units/μL), 2 μL of 2.5 mM dNTP mixture, 1 μL of each primer (forward and reverse), 2.5 μL of 10× DNA buffer, and 2 μL of template DNA. The thermal cycling profile consisted of an initial denaturation at 94 °C for 5 min, followed by 30 cycles of denaturation at 94 °C for 30 s, primer annealing at 50 °C for 40 s, and extension at 72 °C for 1 min, with a final extension step at 72 °C for 7 min. After amplification, PCR products were evaluated by electrophoresis on 1% agarose gels and visualized using GelRed™ Nucleic Acid Gel Stain (Biotium, Fremont, CA, USA). The ITS and *rbcL* amplicons, approximately 653 bp and 1003 bp, were excised and purified using the E.Z.N.A.^®^ Gel Extract kit (Omega Bio-Tek, Inc., Norcross, GA, USA) to remove any potential contaminants or non-specific products. The purified PCR products were then submitted to ATGC Co., Ltd., Khlong Luang, Pathum Thani, Thailand, for DNA sequencing using the Sanger sequencing technique, which provides high-quality sequence data suitable for subsequent phylogenetic and genetic diversity analyses. All sequences generated in the current investigation were deposited in GenBank under the accession numbers PV290460 through PV290476 for ITS sequences and PV295556 through PV295557 for *rbcL* sequences.

### 2.4. Data Analysis

The complete ITS region (comprising partial 18S rRNA, ITS1, 5.8S rRNA, ITS2, and partial 28S rRNA) and *rbcL* sequences obtained in this study were systematically aligned using the ClustalW program (Version 2.1) [19] followed by manual inspection and refinement in the BioEdit sequence alignment editor [20]. This two-step alignment process ensured optimal sequence comparison by combining algorithmic alignment with expert visual verification.

For population genetic analysis, molecular diversity indices and haplotype data were generated using DnaSP v5 software [21], providing critical metrics on genetic variation within and between populations. Genetic differentiation between populations was quantified using both uncorrected *p*-distance and Kimura 2-parameter (K2P) distance metrics [22] as implemented in MEGA (Version 11) [23]. The K2P model was particularly valuable as it accounts for transitional and transversional substitution rate biases commonly observed in DNA sequence evolution. *F*-statistics (*F*_ST_) for the ITS region sequences were calculated using Arlequin v. 3.5 [24]. Following this, gene flow (Nm) was determined using the formula Nm = (1 − *F*_ST_)/4 × *F*_ST_, which was introduced by Wright [25], to measure the extent of gene flow among populations. To visualize the relationships among haplotypes, a minimum-spanning network was constructed using the Network program (version 10.2) based on the median-joining network algorithm [26]. This network representation offered an intuitive visualization of genetic relationships and potential evolutionary pathways among the identified haplotypes of *C. paniculatus* populations.

Phylogenetic relationships were investigated through tree-based analyses of the unique haplotypes identified in both ITS and *rbcL* sequences across all sampled *C. paniculatus* populations. The analysis was strengthened by incorporating additional *Celastrus* species sequences retrieved from GenBank, providing a broader taxonomic context. *Glyptopetalum feddei* (H.Lév.) Ding Hou, represented by both *matK* and ITS sequences, was selected as an appropriate outgroup to root the phylogenetic trees based on its established taxonomic relationship within Celastraceae. For tree construction, two complementary phylogenetic methods were employed: maximum likelihood (ML) using the General Time Reversible model with gamma-distributed rate variation and invariant sites (GTR + G + I) [27] and the distance-based neighbor-joining method [28]. Both analyses were conducted using MEGA (Version 11) [29], with statistical confidence in the resulting tree topologies assessed through 1000 bootstrap replicates. This dual-method approach provided a robust framework for evaluating phylogenetic hypotheses and identifying consistent patterns of evolutionary relationships among the studied taxa.

## 3. Results and Discussion

### 3.1. Genetic Variation and Molecular Diversity Indices

Molecular techniques have revolutionized plant genetic diversity and population structure analyses, offering significant advantages over conventional methods. These modern approaches provide researchers with enhanced efficiency through higher processing speeds and reduced costs, while requiring minimal plant material for analysis. Most importantly, they generate more accurate and reliable genetic diversity information, effectively overcoming the inherent limitations of traditional approaches [10,30,31].

Over the past several decades, researchers have developed and deployed a diverse array of molecular markers for investigating the genetic diversity and population structure in plants. These include RFLP, AFLP, RAPD, SSR, SCAR, ISSR, SNPs, and microsatellites. Additionally, specific genetic regions have proven particularly valuable as markers, including chloroplast maturase K (*matK*), ITS, and *rbcL* regions [9,11,12,13,32,33,34,35]. This progressive development of marker technology has expanded the toolkit available to plant geneticists and taxonomists.

When selecting markers for studies encompassing both inter- and intraspecific variations, a balance between resolution and practical feasibility is often necessary. While markers such as microsatellites and SNPs can offer a high resolution for intraspecific variation, their development typically requires more complex and resource-intensive processes, which may not be feasible for all research scopes. Consequently, established and widely applied markers like the nuclear ITS region and the plastid *rbcL* gene are frequently utilized. In this context, we selected the ITS region for its known variability and the *rbcL* gene as a conservative plastid marker. Although *rbcL* is characterized by a relatively slow evolutionary rate, its inclusion provides a valuable comparative baseline against the more variable nuclear ITS marker. This combination aids in evaluating broader patterns of genetic differentiation and supports phylogenetic inference, proving useful when assessing both inter- and intraspecific patterns [10,11,12,13]. Indeed, these two markers, the ITS and *rbcL*, form integral components of standardized plant DNA barcode systems, enabling a rapid and accurate species identification by analyzing short, standardized genetic sequences. The adoption of these barcoding systems has facilitated more systematic approaches to understanding plant genetic diversity, evolutionary relationships, and population dynamics across diverse ecosystems [10,11,12,13].

In this study, both ITS and *rbcL* markers were employed to conduct a comprehensive genetic analysis of *C. paniculatus* samples collected from the northern and northeastern regions of Thailand, where this plant species is abundantly distributed. The analysis of the 653 bp ITS sequences revealed a nucleotide variation at 17 positions (2.60%), resulting in 17 distinct haplotypes (Table 2). The observed mutations comprised five pyrimidine transitions (positions 16, 34, 194, 420, and 594), two purine transitions (positions 186 and 196), and ten transversions (positions 1, 61, 70, 122, 124, 128, 172, 263, 503, and 520). This diversity in mutation types suggests that multiple evolutionary events have shaped the genetic structure within the studied populations.

In contrast to the ITS region, the 1003 bp *rbcL* sequences showed minimal variation, with only one transversion mutation (0.09%) at position 636. This significantly lower variability indicates that the *rbcL* gene is more conserved than the ITS region in these populations. Our findings align with Mu et al. [11], who also reported a high genetic variation in Chinese *Celastrus* based on the ITS region and a low genetic variation based on *rbcL* sequences.

The molecular diversity analysis revealed significant variations in the genetic diversity across the studied populations of *C. paniculatus* (Table 3), which were similar to previous studies by Mu et al. [11,33] using nuclear (ETS and ITS) and plastid region (*psbA-trnH*, *rbcL*, *rpl16*, and *trnL-F*) markers. This research examined 62 individuals distributed among multiple populations, identifying 17 segregating sites that corresponded to 17 distinct haplotypes (designated CpI1−CpI17), with 15 being unique to specific populations. The CMI population demonstrated the highest level of genetic diversity with seven distinct haplotypes, exhibiting a high haplotype diversity index (Hd = 0.944 ± 0.070) and the highest nucleotide diversity (Nd = 0.0039 ± 0.0007). This suggests that CMI has maintained a robust genetic structure with minimal inbreeding or population bottlenecks.

Similarly, the MKM population showed a substantial genetic variation with seven haplotypes and a high haplotype diversity (Hd = 0.911 ± 0.077), indicating another genetically healthy population with considerable variation. The PLK population exhibited moderate genetic diversity (Hd = 0.556 ± 0.090), while NPM and UBN populations showed relatively lower levels of genetic variation with haplotype diversities of 0.436 ± 0.133 and 0.250 ± 0.180, respectively. These intermediate values suggest that these populations may have experienced some degree of genetic drift or restricted gene flow.

In marked contrast to the diverse populations, the LEI and LPN populations displayed a complete absence of genetic variation (Hd = 0.000 ± 0.000, Nd = 0.0000 ± 0.0000), with each population containing only a single haplotype. This lack of diversity strongly suggests that these populations have experienced severe genetic bottlenecks, founder effects, or prolonged isolation. These findings have significant implications for conservation strategies. The genetically depauperate LEI and LPN populations should be prioritized for genetic rescue interventions to prevent the further loss of adaptive potential. Meanwhile, the genetically diverse CMI and MKM populations could serve as potential sources of genetic material if translocation or breeding programs become necessary. The variation in the genetic diversity across these populations underscores the importance of population-specific conservation approaches rather than implementing a single management strategy across all populations.

### 3.2. Haplotype Network Analysis

**Figure 2:**
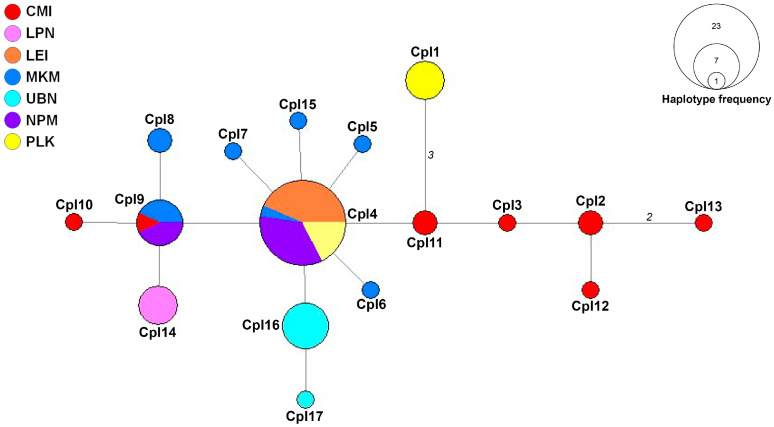
The minimum spanning haplotype network constructed from the ITS haplotypes of *Celastrus paniculatus* from Thailand. Different colors in the haplotype networks represent the various localities examined in this study. The size of each circle reflects the proportion of specimens associated with each haplotype. The length of each branch represents the number of mutational steps (ms), with values greater than one displayed. Locality codes are provided in Table 1.

The haplotype network constructed from ITS sequences revealed a single genetic group with diverse haplotypes distributed across the sampled populations (Figure 2). This network structure suggests an ongoing gene flow between populations while maintaining localized genetic variations. The central haplotype (CpI4) was the most frequent, shared by 23 samples from four populations (MKM, LEI, NPM, and PLK), indicating its potential role as an ancestral or founder haplotype in the region. Similarly, haplotype CpI9 appeared in seven samples across three populations (CMI, MKM, and NPM), further demonstrating the connectivity between these locations.

Population-specific patterns of genetic diversity were evident throughout the network. The CMI population exhibited an exceptionally high genetic variation, harboring multiple distinct haplotypes (CpI2, CpI3, CpI10, CpI12, and CpI13). This observation aligns with the molecular diversity indices, which identified CMI as having the highest haplotype diversity, possibly indicating this area as a center of genetic diversity or an ecological transition zone, such as between the deciduous dipterocarp forest (DDF) and the lower montane forest (LMF). In contrast, the LEI population was primarily associated with the central haplotype (CpI11) and lacked unique haplotypes, confirming its low genetic variation and suggesting a potential founder effect or a recent population establishment. Similarly, LPN demonstrated limited genetic variation with only a single unique haplotype (CpI14), which may reflect either genetic bottlenecks or limited sampling in this region.

The MKM population displayed a well-distributed range of haplotypes (CpI4, CpI6, CpI7, and CpI8), suggesting significant genetic diversity and potentially indicating long-term population stability in this area. The UBN population contained multiple unique haplotypes (CpI16 and CpI17), indicating genetic differentiation that may result from geographic barriers or adaptations to local environmental conditions. While NPM was predominantly linked to the central haplotype (CpI11) with some variation, the PLK population featured a distinct haplotype (CpI1) separated by several mutation steps from other haplotypes. This genetic distance suggests possible geographic isolation, limited gene flow, or divergent selection pressures acting on the PLK population over time. The mutational steps separating CpI1 from the central haplotypes could represent an early stage of allopatric differentiation, warranting further investigation into the ecological and geographical factors influencing this population.

In addition, the unique haplotypes strictly observed in specific populations, particularly haplotype CpI1 found only in PLK and haplotypes CpI16 and CpI17 found exclusively in UBN, should be prioritized for conservation in order to preserve these rare and population-specific genotypes, which may hold important evolutionary, ecological, or adaptive significance.

### 3.3. Genetic Differences and Gene Flow Analyses

The genetic differentiation among the seven populations of *C. paniculatus* was quantified using two complementary distance measures: the K2P model, which accounts for higher rates of transitions versus transversions, and the simpler *p*-distance model, which directly calculates the proportion of nucleotide differences between sequences [22,23]. The analysis revealed a spectrum of genetic relationships among populations (Table 4). Pairwise genetic distances calculated using the *p*-distance model ranged from 0.0004 to 0.0065 (0.04% to 0.65% sequence divergence), while the K2P distances showed similar but slightly different values, ranging from 0.0004 to 0.0059 (0.04% to 0.59%). The congruence between these two distance metrics supports the observed genetic differentiation.

The populations of LEI and NPM exhibited the lowest genetic distance (0.0004 for both *p*-distance and K2P models), indicating a remarkably high genetic similarity. This close relationship suggests recent connectivity, possible migration events, or a shared ancestry between these geographically distinct populations. The near-identical genetic composition might indicate that these populations have experienced similar selective pressures or have maintained the gene flow despite the geographical separation. In contrast, several population pairs displayed substantially higher genetic divergence (*p*-distance and K2P values exceeding 0.005). Most notably, the PLK population showed a significant differentiation when compared to CMI, LPN, MKM, and UBN populations. Similarly, the CMI population exhibited a considerable genetic distance from both MKM and UBN populations. These elevated divergence values suggest either long-term isolation, an adaptation to different environmental conditions, or historical separation events that have limited gene flow. The observed patterns of genetic diversity parallel those found in other plant species such as *Liriodendron chinense* [36].

The pattern of genetic distances across the study area suggests a complex evolutionary history for *C. paniculatus* in Thailand, potentially reflecting the influence of geographical barriers, habitat fragmentation, or climatic fluctuations on the population connectivity and genetic exchange. These findings provide important baseline information for conservation strategies, particularly for identifying evolutionarily significant units within the species [10].

In addition, we analyzed *F*_ST_ and Nm values to assess the genetic differentiation and estimate the gene flow among populations. *F*_ST_ values ranged from 0.0302 to 1.0000, while Nm values ranged from 0.0000 to 8.0364 (Table 5). Based on the *F*_ST_ analysis, all population comparisons showed significant differences except between NPM and LEI (*F*_ST_ = 0.1859) and between NPM and MKM (*F*_ST_ = 0.0302).

**Figure 3:**
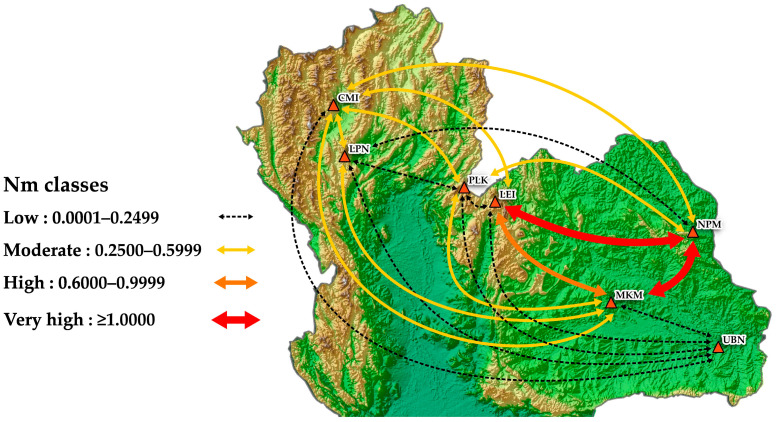
Comparative level of gene flow between populations of *Celastrus paniculatus* in Thailand based on Nm value, classified into four classes.

The gene flow estimation based on Nm values was categorized into four classes (Figure 3): very high (≥1.0000), high (0.6000–0.9999), moderate (0.2500–0.5999), and low (0.0000–0.2499). A low to moderate gene flow was observed between most populations. For instance, a low gene flow was found between UBN and MKM, LIE, PLK, LPN, and CMI, while a moderate gene flow was observed between CMI and LPN, LIE, PLK, MKM, and NPM (Figure 3). A high gene flow was recorded between LEI and MKM (Nm = 0.8750). A very high gene flow was detected between LEI and NPM (Nm = 1.0945), with the highest gene flow observed between NPM and MKM (Nm = 8.0364). These findings indicated that *C. paniculatus* populations in Thailand largely lack gene flow. Therefore, ex situ planting or the establishment of seed banks may be effective methods for further conserving the genetic resources of this plant throughout Thailand and its endemic areas.

### 3.4. Phylogenetic Tree Analysis

**Figure 4:**
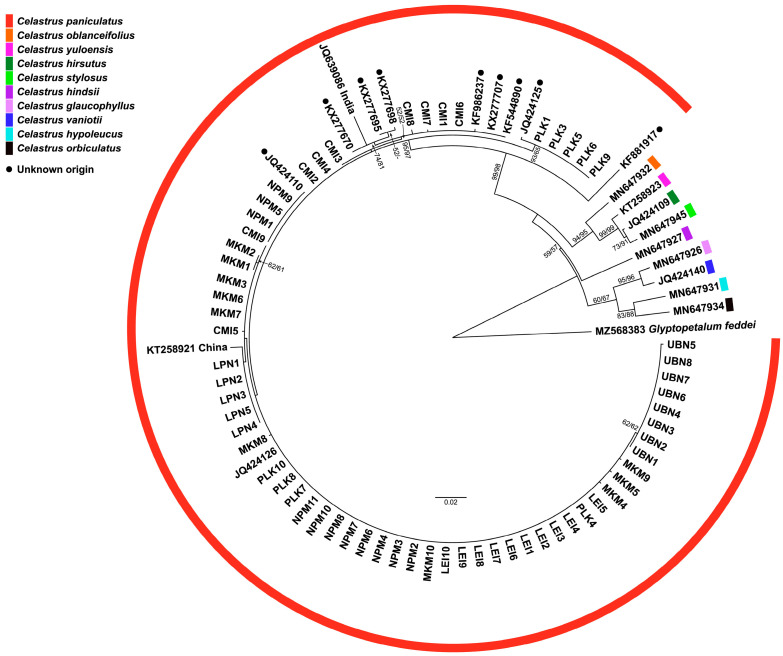
A phylogenetic tree based on ITS sequences showing relationships among *Celastrus paniculatus* samples from this study and previously published sequences of *C. paniculatus* and other *Celastrus* species from GenBank. Numbers at nodes indicate bootstrap support values from maximum likelihood/neighbor-joining analyses. *Glyptopetalum feddei* was used as an outgroup.

The phylogenetic analyses using two different genetic markers provided complementary insights into the evolutionary relationships among the studied *C. paniculatus* populations in Thailand. The phylogenetic tree constructed using the nuclear ribosomal DNA region (partial 18S rRNA, ITS1, 5.8S rRNA, ITS2, and partial 28S rRNA sequences) revealed well-defined hierarchical relationships among the populations (Figure 4). This nuclear marker, known for its faster evolutionary rate, captured recent divergence patterns effectively [9,13,37].

Most individuals from PLK, LPN, MKM, NPM, and CMI formed a strongly supported monophyletic clade, indicating close genetic relationships and suggesting a shared evolutionary history. This clustering pattern suggests either a recent population divergence or the maintenance of genetic connectivity through an ongoing gene flow across most of the sampled range. The strong monophyly of this primary clade supports the taxonomic integrity of *C. paniculatus* in Thailand as a cohesive evolutionary unit. Within this main clade, several distinct subclades emerged with varying levels of support. Notably, individuals from the PLK population formed a distinct group, confirming the genetic divergence suggested by the haplotype network analysis. This genetic differentiation could be attributed to geographical isolation, ecological specialization, or historical vicariance events that limited the gene flow between PLK and other populations.

Interestingly, the LPN population, together with a reference sequence from China, formed a moderately supported subclade nested within the primary Thai clade. This positioning suggests historical connectivity between Thai and Chinese populations, potentially indicating past migration corridors or a broader historical distribution range for the species. The intermediate phylogenetic position of the LPN population might represent an evolutionary transition zone between Thai and Chinese lineages [11].

In contrast to the Thai populations, reference sequences from India and several GenBank accessions formed distinct lineages separated by longer branch lengths, indicating substantial genetic divergence. This pattern suggests long-term geographical isolation or adaptive divergence between the Indian and Thai populations, potentially approaching species-level differentiation. The estimated divergence time between these lineages, based on molecular clock analyses of the ITS region, likely corresponds to Pleistocene climate fluctuations that altered forest distributions across Southeast Asia [32].

**Figure 5:**
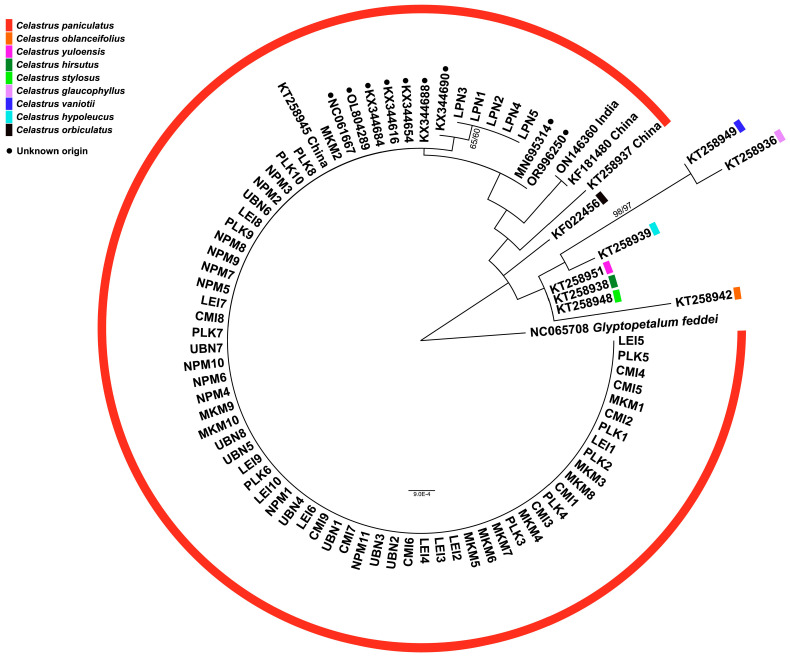
A phylogenetic tree based on *rbcL* sequences showing relationships among *Celastrus paniculatus* samples from this study and previously published sequences of *C. paniculatus* and other *Celastrus* species from GenBank. Numbers at nodes indicate bootstrap support values from maximum likelihood/neighbor-joining analyses. *Glyptopetalum feddei* was used as an outgroup.

The phylogenetic reconstruction based on the chloroplast *rbcL* gene (Figure 5) largely corroborated the ITS-based findings while revealing additional evolutionary patterns. The *rbcL* gene, evolving more slowly than ITS regions, typically reflects deeper evolutionary relationships and maternal lineage history [38,39]. The majority of individuals from PLK, CMI, MKM, NPM, and LEI formed a well-supported monophyletic clade, confirming their close genetic relationship inferred from ITS data. The strong clustering of these populations in both nuclear and chloroplast markers suggests concordant evolutionary histories with a limited impact from hybridization or introgression events.

Notably, the *rbcL* phylogeny revealed that individuals from LPN formed a separate, well-supported cluster, demonstrating greater genetic divergence than observed in the ITS phylogeny. This discordance between nuclear and chloroplast markers could indicate the historical chloroplast capture through hybridization, lineage sorting effects, or different selective pressures acting on these genomic regions. The observed patterns of the phylogenetic analysis parallel those reported in Chinese Celastrus [11,33]. Notably, the distinct positioning of LPN in the *rbcL* phylogeny further supports the hypothesis that this population represents a genetically distinct lineage, warranting conservation attention.

Reference sequences from other *Celastrus* species formed phylogenetically distinct clades in both analyses, confirming the taxonomic integrity of *C. paniculatus* as a valid species. The systematic relationships revealed by both markers also demonstrated the close affinity of certain Thai populations with reference sequences from China and more distant relationships with Indian lineages, suggesting an east-to-west pattern of the historical dispersal and differentiation across the species’ range in Asia. The congruent yet complementary patterns revealed by these two independent genetic markers provide robust evidence for the evolutionary history and population structure of *C. paniculatus* in Thailand, offering valuable insights for taxonomy, biogeography, and conservation planning.

The genetic variation and haplotype network analysis provide robust support for the molecular diversity findings, revealing a complex genetic landscape among the studied *C. paniculatus* populations in Thailand. Populations such as CMI and MKM exhibited a remarkably high genetic diversity, characterized by numerous distinct haplotypes and high haplotype diversity indices. This genetic richness suggests these areas may represent long-established populations or potential refugia that their maintained diversity through historical climate fluctuations. In contrast, LEI and LPN populations demonstrated limited genetic variation, possibly indicating recent founder events, genetic bottlenecks, or edge-of-range effects that have constrained their genetic diversity. The observed genetic structure reflects a dynamic evolutionary history, balancing a shared ancestry with localized differentiation. The central position and high frequency of haplotype CpI4 across multiple populations suggest it likely represents an ancestral lineage from which other haplotypes have diverged. This pattern of the star-like radiation from a common ancestor is characteristic of recent population expansions following historical bottlenecks, possibly associated with post-glacial forest recolonization events in Southeast Asia.

Notably, UBN and PLK populations harbored multiple private haplotypes (haplotypes exclusive to a single population), indicating a significant genetic uniqueness. This pattern strongly suggests a degree of reproductive isolation and limited gene flow with other populations, potentially due to geographical barriers, phenological differences, or local adaptations to specific ecological niches. These populations may represent distinct evolutionary units worthy of special conservation attention as they contain unique genetic variations not found elsewhere within the range of the species. The widespread distribution of haplotype CpI11 across geographically distant populations provides compelling evidence for historical connectivity or ancient dispersal events that established a common genetic foundation throughout the region. This shared genetic lineage might reflect historical migration corridors that facilitated gene flow during periods of forest continuity before fragmentation created the current population structure.

Future research should integrate these genetic findings with ecological and environmental data to explore the potential adaptive divergence among populations. Expanding the sampling to include populations throughout the various distribution localities worldwide would help establish a comprehensive phylogeographic framework for understanding the species’ evolutionary history across its entire range. Genome-wide approaches, such as restriction site-associated DNA sequencing (RAD-seq) or whole-genome resequencing, would provide a higher resolution for detecting fine-scale population structures and signatures of selection [12,38,39,40]. Additionally, studies incorporating paleoclimatic modeling could help reconstruct historical distribution patterns and identify potential refugia that shaped the current genetic diversity [36,41]. Conservation efforts should prioritize populations with high genetic diversity (CMI and MKM) as reservoirs of adaptive potential, while also protecting genetically distinct populations (UBN and PLK) to preserve unique evolutionary lineages. Finally, reciprocal transplant experiments could test for local adaptations among genetically divergent populations, providing insights into the ecological drivers of genetic differentiation and informing conservation management strategies in the face of ongoing climate change.

## 4. Conclusions

The genetic analysis of *C. paniculatus* populations across northern and northeastern Thailand revealed complex evolutionary patterns and meaningful insights into the species’ population structure. Using nuclear ITS and chloroplast *rbcL* markers, this study identified a significant variation in the genetic diversity among the seven studied populations. The CMI and MKM populations exhibited a remarkably high genetic diversity with numerous distinct haplotypes, suggesting these areas may represent historical refugia or long-established populations. In contrast, LEI and LPN demonstrated limited genetic variation, potentially indicating genetic bottlenecks or recent founder events. The haplotype network analysis revealed a star-like pattern radiating from central haplotype CpI4, indicating a common ancestral lineage characteristic of a recent population expansion. UBN and PLK populations harbored multiple private haplotypes, suggesting a limited gene flow due to geographical isolation or local adaptation. The widespread distribution of haplotype CpI11 provided evidence for historical connectivity throughout the region. Phylogenetic analyses confirmed the taxonomic integrity of *C. paniculatus* while revealing population-specific clustering patterns. Genetic distance analyses showed LEI and NPM with the closest relationship (0.0004 for both *p*-distance and K2P), while PLK displayed a significant differentiation from other populations. These findings establish a foundation for effective in situ conservation strategies for this valuable medicinal plant, highlighting the importance of preserving both genetically diverse and distinct populations. Furthermore, these insights should inform future ex situ conservation efforts, including the strategic development of seed banks and living collections to safeguard populations with a high diversity or unique private haplotypes, thereby ensuring the long-term preservation of the species’ overall genetic heritage and evolutionary capacity.

## Figures and Tables

**Table 1:** Sampling localities and details of *Celastrus paniculatus* collected in this study.

Code	*N*	District	Province	Region	Altitude (m)	Latitude	Longitude
CMI	9	Mae Rim	Chiang Mai	North	776	18.889889	98.860825
PLK	9	Chat Trakan	Phitsanulok	North	1066	17.608864	100.902396
LPN	5	Thung Hua Chang	Lamphun	North	840	18.097083	99.033806
LEI	10	Phu Ruea	Loei	Northeast	653	17.391944	101.370556
MKM	10	Na Chuek	Maha Sarakham	Northeast	167	15.836415	103.159919
NPM	11	Na Kae	Nakhon Phanom	Northeast	140	16.926970	104.438570
UBN	8	Warinchamrab	Ubon Ratchathani	Northeast	120	15.139028	104.832361
Total	62						

*N*, sample size.

**Table 2:** Variable nucleotide positions compared between different ITS haplotypes of *Celastrus paniculatus* in Thailand.

Haplotypes	Nucleotide Variable Positions of ITS Region																
						1	1	1	1	1	1	1	2	4	5	5	5
		1	3	6	7	2	2	2	7	8	9	9	6	2	0	2	9
	1	6	4	1	0	2	4	8	2	6	4	6	3	0	3	0	4
CpI1	A	C	C	C	C	C	A	A	C	A	C	G	G	T	T	G	C
CpI2	.	.	T	.	.	.	.	.	.	G	T	.	.	C	G	.	.
CpI3	.	.	T	.	.	.	.	.	.	G	T	.	.	.	G	.	.
CpI4	.	T	.	.	.	.	.	.	.	G	T	.	.	.	G	.	.
CpI5	.	T	.	G	.	.	.	.	.	G	T	.	.	.	G	.	.
CpI6	.	T	.	.	G	.	.	.	.	G	T	.	.	.	G	.	.
CpI7	C	T	.	.	.	.	.	.	.	G	T	.	.	.	G	.	.
CpI8	.	T	.	.	.	A	T	.	.	G	T	.	.	.	G	.	.
CpI9	.	T	.	.	.	A	.	.	.	G	T	.	.	.	G	.	.
CpI10	.	T	.	.	.	A	.	.	.	G	T	.	.	C	G	.	.
CpI11	.	.	.	.	.	.	.	.	.	G	T	.	.	.	G	.	.
CpI12	.	.	T	.	.	.	.	.	.	G	T	A	.	C	G	.	.
CpI13	.	.	T	.	.	.	.	.	.	G	T	.	C	C	G	C	.
CpI14	.	T	T	.	.	A	.	.	.	G	T	.	.	.	G	.	.
CpI15	.	T	.	.	.	.	.	C	.	G	T	.	.	.	G	.	.
CpI16	.	T	.	.	.	.	.	.	A	G	T	.	.	.	G	.	.
CpI17	.	T	.	.	.	.	.	.	A	G	T	.	.	.	G	.	T

**Table 3:** Molecular diversity indices of *Celastrus paniculatus* from different geographical localities in Thailand based on ITS sequences analysis.

Populations	Molecular Diversity Indices					
	n	S	H	Uh	Hd ± SD	Nd ± SD
PLK	9	4	2	1	0.556 ± 0.090	0.0034 ± 0.0006
CMI	9	7	7	6	0.944 ± 0.070	0.0039 ± 0.0007
LEI	10	0	1	0	0.000 ± 0.000	0.0000 ± 0.0000
MKM	10	6	7	5	0.911 ± 0.077	0.0026 ± 0.0004
LPN	5	0	1	1	0.000 ± 0.000	0.0000 ± 0.0000
NPM	11	1	2	0	0.436 ± 0.133	0.0007 ± 0.0002
UBN	8	1	2	2	0.250 ± 0.180	0.0004 ± 0.0003
**Total**	**62**	**17**	**17**	**15**	**0.832 ± 0.039**	**0.0032 ± 0.0004**

n, sample size; S, segregation site; H, number of haplotypes; Uh, unique haplotype; Hd, haplotype diversity; Nd, nucleotide diversity; and SD, standard deviation. Locality codes are provided in Table 1.

**Table 4:** Genetic differences K2P (lower triangle) and *p*-distance (upper triangle) calculated based on ITS (lower triangle) and compared among different populations of *Celastrus paniculatus*.

Codes *	CMI	LPN	LEI	MKM	UBN	NPM	PLK
**CMI**	–	0.0044	0.0037	0.0051	0.0055	0.0040	0.0058
**LPN**	0.0044	–	0.0031	0.0032	0.0048	0.0026	0.0065
**LEI**	0.0038	0.0031	–	0.0017	0.0017	0.0004	0.0034
**MKM**	0.0051	0.0032	0.0017	–	0.0034	0.0017	0.0051
**UBN**	0.0055	0.0048	0.0017	0.0034	–	0.0021	0.0051
**NPM**	0.0040	0.0027	0.0004	0.0017	0.0021	–	0.0038
**PLK**	0.0059	0.0065	0.0034	0.0051	0.0052	0.0038	–

* Code of populations is provided in more detail in Table 1.

**Table 5:** The *F*-statistics (*F*_ST_) (lower triangle) and gene flow (Nm) between the seven different populations of *Celastrus paniculatus* in Thailand based on the ITS sequence analysis.

Codes *	PLK	CMI	LEI	MKM	LPN	NPM	UBN
**PLK**	-	0.4225	0.2326	0.3591	0.1233	0.2596	0.1552
**CMI**	0.3718	-	0.2544	0.4415	0.2862	0.3069	0.1721
**LEI**	0.5181	0.4956	-	0.8750	0.0000	1.0945	0.0273
**MKM**	0.4104	0.3615	0.2222	-	0.2570	8.0364	0.2187
**LPN**	0.6697	0.4662	1.0000	0.4931	-	0.0525	0.0133
**NPM**	0.4906	0.4489	*0.1859*	*0.0302*	0.8264	-	0.0855
**UBN**	0.6169	0.5923	0.9017	0.5333	0.9495	0.7451	-

* Code of populations is provided in more detail in Table 1. Italicized values indicate non-significant results (*p* ≥ 0.05).

## Data Availability

The original contributions presented in this study are included in the article. Further inquiries can be directed to the corresponding author.
